# Aripiprazole- and Lurasidone-Induced Akathisia: A Case Report and Literature Review

**DOI:** 10.7759/cureus.92704

**Published:** 2025-09-19

**Authors:** Kalyan Kandra

**Affiliations:** 1 Psychiatry, Southern Illinois University School of Medicine, Springfield, USA

**Keywords:** akathisia, akathisia treatment, antipsychotics, aripiprazole, extrapyramidal symptoms, lurasidone, movement disorders

## Abstract

Akathisia is a distressing extrapyramidal symptom of antipsychotic medications, characterized by a subjective feeling of inner restlessness and an objective urge to move. While first-generation antipsychotics are often associated with akathisia, second-generation antipsychotics, including partial dopamine agonists, such as aripiprazole and serotonin-dopamine activity modulators like lurasidone, may also cause akathisia. This report details the case of a 46-year-old male with treatment-resistant depression who developed severe akathisia sequentially during augmentation therapy, first with lurasidone and later with aripiprazole. In both instances, the distressing symptoms resolved completely upon discontinuation of the offending agent. This case is significant as it highlights that even low, introductory doses of these commonly used second-generation antipsychotics can precipitate severe akathisia. It underscores the critical need for vigilant clinical monitoring and prompt intervention to prevent patient distress and ensure appropriate management, particularly in complex cases like treatment-resistant depression.

## Introduction

Antipsychotic medications are commonly prescribed for the treatment of various psychiatric disorders, including schizophrenia, bipolar disorder, and major depressive disorder [1-3]. However, their therapeutic benefits are often accompanied by various adverse effects, including extrapyramidal symptoms (EPS) [4,5]. These movement disorders typically emerge in a temporal sequence following initiation or dose escalation of an antipsychotic. Acute dystonia, characterized by sudden, involuntary muscle spasms, often appears first within hours to days. This can be followed by akathisia within days to weeks, and drug-induced Parkinsonism (tremor, rigidity, and bradykinesia) within weeks to months. Tardive dyskinesia, a potentially irreversible syndrome of involuntary, repetitive movements, is a late-onset complication that emerges after months or years of therapy [4,6].

Akathisia, derived from the Greek meaning "not to sit," is a particularly troublesome EPS characterized by a subjective sensation of inner restlessness and an objective urge to move, leading to repetitive, semi-purposeful movements, such as pacing, rocking, or shifting weight [6]. The prevalence of antipsychotic-induced akathisia can be as high as 20-30% with first-generation antipsychotics (FGAs) and, while generally lower with second-generation antipsychotics (SGAs), remains a significant clinical concern, with reported rates of 15-25% for partial dopamine agonists like aripiprazole [7,8]. Untreated or misdiagnosed akathisia can lead to severe distress, poor medication adherence, treatment resistance, and an increased risk of suicidal ideation and aggressive behavior [9]. Distinguishing akathisia from agitation, anxiety, or primary psychotic symptoms is crucial for appropriate management [6]. This report details a challenging case of akathisia experienced by a patient with treatment-resistant depression (TRD) treated sequentially with lurasidone and aripiprazole, providing a detailed account of its presentation, diagnostic considerations, and management. Accompanying this case is a comprehensive review on antipsychotic-induced akathisia, with particular emphasis on partial dopamine agonists and their unique pharmacological profiles.

## Case presentation

Patient information

This patient is a 46-year-old male with a long-standing diagnosis of treatment-resistant depression (TRD), who presented to the outpatient psychiatric clinic for augmentation of his antidepressant regimen. His current antidepressant was 40 mg of vilazodone. He had a history of trying several selective serotonin reuptake inhibitors (SSRIs), serotonin-norepinephrine reuptake inhibitors (SNRIs), norepinephrine dopamine reuptake inhibitors (NDRIs), tricyclic antidepressants (TCAs), and lithium in the past, none of which provided adequate relief for his TRD. He was also on a weekly esketamine 84 mg dose regimen. His medical history was otherwise unremarkable, with no history of substance use disorder or pre-existing movement disorders. The patient did not receive electroconvulsive therapy (ECT) and was never on clozapine.

To augment his ongoing vilazodone 40 mg daily, he was initially started on lurasidone 20 mg daily. Approximately two weeks after initiating lurasidone, he began experiencing subjective feelings of "inner jitters" and an "unbearable urge to keep moving his legs," particularly when attempting to sit still. He described constantly shifting in his chair and pacing a lot. Objectively, prominent motor restlessness was observed, including frequent leg movements, rocking, and a marked inability to maintain a stationary posture. His Barnes Akathisia Rating Scale (BARS) score at this time was 4, indicating definite subjective and objective akathisia with moderate distress. Due to the emergent and distressing nature of the akathisia, lurasidone was immediately discontinued. Following its discontinuation, his akathisia symptoms resolved completely within a few days without the need for any specific pharmacological treatment, and his BARS score returned to 0.

Given his continued residual depressive symptoms, bupropion/dextromethorphan 45-105 mg twice daily was subsequently added to his regimen (alongside vilazodone and weekly esketamine). While he experienced some partial improvement in his depressive symptoms with this combination, residual symptoms persisted, necessitating further augmentation. As an augmentation, aripiprazole 2 mg was initiated daily. Approximately one week after starting aripiprazole, he again reported the return of severe "inner restlessness" and a compelling need to move, symptoms strikingly reminiscent of his previous experience with lurasidone. Clinically, constant fidgeting and an inability to remain seated were observed, leading to a BARS score of 5 (severe subjective and objective akathisia with severe distress). He also reported heightened anxiety and dysphoria directly associated with akathisia. Aripiprazole was immediately discontinued. Following its withdrawal, his akathisia symptoms began to subside over the next few days and were resolved completely, again without the need for any specific pharmacological intervention for akathisia. His BARS score returned to 0, and alternative augmentation strategies for his TRD were then considered. Table 1 outlines the patient's medication changes and the resulting clinical events.

**Table 1:** Clinical course chart of the patient. BARS: Barnes Akathisia Rating Scale

Time point	Medication change	Clinical event/observation	BARS score
Baseline	On vilazodone 40 mg	The patient has treatment-resistant depression (TRD)	0
Day 0	Lurasidone 20 mg initiated	Augmentation for TRD	-
~Week 2	-	Akathisia develops ("inner jitters," pacing)	4 (moderate)
~Week 2+	Lurasidone discontinued	Action taken due to distressing akathisia	-
Following days	-	Akathisia symptoms completely resolve	0
Interim	Bupropion/dextromethorphan added	New medication tried for TRD	0
New baseline	Aripiprazole 2 mg initiated	Further augmentation for residual symptoms	-
~Week 1	-	Severe akathisia returns (restlessness, anxiety)	5 (severe)
~Week 1+	Aripiprazole discontinued	Action taken due to severe akathisia	-
Following days	-	Akathisia symptoms completely resolve again	0

Diagnostic assessment

The diagnosis of antipsychotic-induced akathisia was made based on the clear temporal relationship between the initiation of lurasidone and aripiprazole and the rapid onset/exacerbation of subjective and objective restlessness. Other causes of restlessness, such as anxiety, agitation related to depression, or substance withdrawal, were ruled out through careful clinical evaluation and history taking. The worsening of symptoms with exposure to these specific antipsychotics and subsequent resolution upon their discontinuation, without the need for additional anti-akathisia medication, further supported the diagnosis. The BARS was instrumental in quantifying the severity and monitoring the response to interventions.

Therapeutic intervention and follow-up

Akathisia significantly impacted the patient's daily functioning. He reported feeling intensely restless and tense, which prevented him from engaging in his usual activities, including watching TV, playing video games, or spending time with family. This internal and external agitation was emotionally disturbing and led to his unwillingness to continue the antipsychotic medications. The primary interventions involved promptly discontinuing the offending agents (lurasidone and aripiprazole) upon the emergence of akathisia. Notably, his akathisia symptoms resolved spontaneously after discontinuing the respective antipsychotics, without the need for additional pharmacological treatment (e.g., beta-blockers or benzodiazepines) specifically for akathisia. Subsequent follow-up demonstrated that his akathisia was fully resolved. His treatment-resistant depression required further careful consideration of augmentation strategies with agents known to have lower akathisia risk profiles.

## Discussion

This study highlights the complex and often challenging nature of antipsychotic-induced akathisia. Akathisia is a complex neuropsychiatric syndrome with both subjective and objective manifestations [1,2]. The subjective component involves an unpleasant sensation of internal uneasiness or a compelling urge to move, while the objective component manifests as observable motor restlessness like pacing or an inability to sit still [10]. It is classified into acute, tardive, and withdrawal types based on its temporal course [3]. While the precise pathophysiology is not fully understood, it is primarily linked to the blockade of dopamine D2 receptors in the mesolimbic and nigrostriatal pathways [4]. This blockade can lead to a compensatory increase in dopamine turnover, potentially contributing to akathisia [4,5]. Standardized rating scales, such as the Barnes Akathisia Rating Scale (BARS), are invaluable for assessing severity and monitoring the response to interventions [6].

To provide a broader context for this case, a targeted literature review on "antipsychotic-induced akathisia" was conducted. This review encompassed various studies, including early case reports on aripiprazole [11,12] and risperidone [13], broad reviews on the clinical features and pathophysiology of akathisia [14,10], systematic analyses of treatment options [15,16], and emerging research in pharmacogenomics (Table 2) [17].

**Table 2:** Targeted literature review on antipsychotic-induced akathisia. AIA: antipsychotic induced akathisia; SGA: second generation antipsychotic; RCT: randomized controlled trial; MDD: major depressive disorder

Studies	Study type/population	Drug(s)	Key findings
Basu and Brar (2006)	Case report	Aripiprazole	Dose-dependent rapid-onset akathisia in schizoaffective disorder.
Hettema and Ross (2007)	Case report	Aripiprazole	Tardive akathisia improved with ropinirole.
Shimizu et al. (2002)	Case report	Risperidone + levomepromazine	Delayed-onset nocturnal akathisia reported.
Salem et al. (2017)	Narrative review	Antipsychotics (general)	Incidence, mechanisms, and treatment challenges reviewed.
Marcus et al. (2008)	Multicenter RCT in MDD (augmentation)	Aripiprazole	Demonstrated efficacy but notable akathisia rates.
Jethwa (2015)	Evidence-based algorithm	General AIA	ß-blockers first-line; benzodiazepines/mirtazapine as adjuncts.
Nasyrova et al. (2023)	Systematic review (pharmacogenomics)	General AIA	SNP biomarkers may predict akathisia susceptibility.
Thomas et al. (2015)	Meta-analysis of RCTs in schizophrenia	Aripiprazole, asenapine, lurasidone	AIA risk doubled vs. placebo; lurasidone highest risk.
Marcus et al. (2008)	RCT in MDD (augmentation)	Aripiprazole	Adjunctive therapy: akathisia in 23.1% vs. 4.5% placebo.
Hsu et al. (2018)	RCT secondary analysis in MDD	Aripiprazole	AIA incidence 26.7% vs. 12.2%; risk linked to baseline severity.
Stroup and Gray (2018)	Narrative review (World Psychiatry)	Antipsychotics (general)	Practical strategies for side effect management.
Pringsheim et al. (2018)	National guideline + systematic review	General AIA	BARS, dose reduction/switch, propranolol, clonazepam, mirtazapine.
Wu et al. (2023)	Dose-response analysis	Multiple SGAs	Higher doses strongly linked to increased AIA risk.
Javed et al. (2019)	Practical guidance review	Lurasidone	AIA incidence ~12.9% vs. 3% placebo; highest at initiation.
Gerolymos et al. (2024)	Network meta-analysis	Multiple interventions	Propranolol and mirtazapine most effective treatments.
Praharaj et al. (2015)	RCT + meta-analysis	Mirtazapine	Low-dose mirtazapine effective for acute akathisia.

The patient's sequential development of akathisia aligns with the known pharmacological profiles of the offending agents. Lurasidone, a second-generation antipsychotic, acts as an antagonist at dopamine D2 and serotonin 5-HT2A receptors, and a partial agonist at 5-HT1A receptors. Despite a favorable metabolic profile, it has been associated with a higher risk of akathisia compared to some other SGAs [18,19]. The patient's subsequent reaction to aripiprazole further underscores this risk. Partial dopamine agonists like aripiprazole have a unique profile, acting as D2 partial agonists to stabilize dopaminergic activity [4]. However, this partial agonism can paradoxically induce akathisia, particularly at higher doses or in susceptible individuals. It is hypothesized that this may lead to relative hypodopaminergic states or that rapid fluctuations in D2 receptor occupancy could contribute to restlessness [4]. Despite their generally improved side effect profiles compared to FGAs, these agents can still precipitate severe akathisia, underscoring the need for vigilant monitoring, especially in sensitive patients [20].

The diagnosis of akathisia relies heavily on clinical observation and patient self-report of subjective internal restlessness, as well as objective motor fidgeting [1]. The rapid onset and severe nature of akathisia experienced by the patient underscore the importance of thorough clinical assessment. It is crucial to differentiate akathisia from other conditions such as anxiety, agitation, restless legs syndrome, or tardive dyskinesia [1]. The key distinction is that restlessness in akathisia is a primary physical urge to move that causes emotional distress, whereas motor agitation in anxiety results from a primary psychological state of worry or fear. Misinterpreting akathisia as worsening anxiety, particularly in the context of TRD, can lead to inappropriate dose increases of the antipsychotic, thereby worsening the condition and increasing patient distress. The subjective nature of akathisia often makes it difficult for patients to articulate, emphasizing the need for clinicians to actively inquire about inner restlessness and observe for objective signs. Standardized rating scales, such as the BARS, are invaluable for assessing severity and monitoring the response to interventions [6].

Several factors can increase the risk of developing antipsychotic-induced akathisia. These include the antipsychotic type and dose, with higher doses and faster titration rates of D2 blocking agents like aripiprazole and lurasidone posing a greater risk [4,21]. Concomitant medications affecting dopamine or serotonin systems, such as SSRIs, can also induce or exacerbate akathisia [10]. Patient characteristics like female sex, pre-existing mood disorders, and iron deficiency are potential risk factors, as are individual variations in drug metabolism related to Cytochrome P-450 enzymes [1,9,17].

Although second-generation antipsychotics (SGAs) generally have a lower risk profile than older agents, the incidence of akathisia remains a significant clinical issue. For aripiprazole, the risk is notably high when used as an adjunctive therapy for major depressive disorder, with clinical trial data showing an incidence of approximately 25% [7,8]. The incidence of lurasidone is also considerable, with studies reporting rates of about 13% in patients with schizophrenia [19]. These figures highlight that even at standard or low doses, newer antipsychotics pose a substantial risk for inducing this highly distressing movement disorder.

The management of antipsychotic-induced akathisia typically involves a stepped approach [15,16]. The first-line strategy, if clinically feasible, is to reduce the dose of the offending antipsychotic or switch to an agent with a lower propensity for akathisia, such as quetiapine or clozapine [22]. In this case, the management approach of promptly discontinuing the offending agent led to the spontaneous resolution of symptoms, which is a noteworthy outcome. This highlights that for some patients, immediate withdrawal of the culprit medication may be sufficient, especially when akathisia is recognized early. This also highlights the crucial role of careful medication selection and titration in vulnerable patients. When discontinuation is not feasible, pharmacological interventions are used. Propranolol is often considered a first-line treatment for its ability to reduce both subjective and objective symptoms [15,23]. Benzodiazepines can provide short-term symptomatic relief, while anticholinergic agents are generally less effective for akathisia but may be considered if concomitant Parkinsonism is present [1]. Low-dose mirtazapine has also shown promise in treating akathisia, while evidence for other agents, such as clonidine and vitamin B6, is limited [24,25].

Future research could explore the role of pharmacogenomics in predicting individual susceptibility to antipsychotic-induced akathisia, potentially allowing for more personalized prescribing strategies, particularly for patients with TRD requiring augmentation [17]. Further understanding of the nuances of partial dopamine agonism and its impact on akathisia, as well as the unique mechanisms of agents like lurasidone, could lead to the development of antipsychotics with even lower akathisia profiles suitable for TRD [4,19].

## Conclusions

Antipsychotic-induced akathisia remains a challenging adverse effect, even with newer second-generation agents like lurasidone and aripiprazole, particularly when used for augmentation in treatment-resistant depression. This study underscores the significant distress and functional impairment that akathisia can cause, emphasizing the critical need for vigilant monitoring, early diagnosis, and prompt intervention. While both lurasidone and aripiprazole carry a notable risk of akathisia, individual patient susceptibility and specific drug properties play a crucial role. In this case, prompt discontinuation of the offending agents led to spontaneous resolution of akathisia symptoms. This report contributes to the growing body of literature on antipsychotic-induced akathisia, reinforcing the importance of a thorough understanding of its pathophysiology and effective management strategies to optimize patient outcomes and adherence to essential psychiatric treatments for complex conditions like TRD. It is important that clinicians monitor patients closely for emergent motor side effects, as severe akathisia can occur even at low, introductory doses of antipsychotics.
